# The unbalanced needs and perceived supply of supportive care in family caregivers of patients with multiple myeloma: a cross-sectional study

**DOI:** 10.1186/s12904-026-02312-9

**Published:** 2026-08-29

**Authors:** Yanfang Lin, Xueting Zhuang, Chunfeng Wang, Chengyang Li, Chunrong Chen, Yong Wu, Rong Hu

**Affiliations:** 1 The School of Nursing, Fujian Medical University, No. 1 Xueyuan Road, Shangjie Town, Minhou County, Fuzhou City, Fujian Province 350108 China; 2 Department of Hematology Nursing, Fujian Medical University Union Hospital, 29 Xinquan Road, Fuzhou, Fujian Province 350001 China; 3 Department of Hematology, Fujian Medical University Union Hospital, 29 Xinquan Road, Fuzhou, Fujian Province 350001 China

**Keywords:** Supportive care, Unmet needs, Caregiving burden, Multiple myeloma

## Abstract

**Background:**

Understanding the needs of family caregivers of patients with cancer and providing appropriate support are essential to ease the burden of illness, facilitate family management, and reduce the risk of adverse health outcomes. Currently, the unmet supportive care needs in family caregivers of patients with multiple myeloma remain unclear.

**Methods:**

A cross-sectional study was conducted. We used a self-developed questionnaire to assess the supportive care needs and perceived supply among 227 family caregivers of patients with multiple myeloma in southeastern China. Multiple regression analysis was performed to identify associated factors.

**Results:**

The highest need was disease information (need rate = 90.04%), and the lowest perceived supply was psychosocial resources (supply rate = 50.05%). Unmet needs span all domains and items. Among the top 10 unmet needs were four items for psychosocial resources, three items for caregiving skills, two items for medical services, and one item for disease information. Caregivers whose patients had rural medical insurance, whose patients were in the primary or consolidation treatment stage, who were spouses of the patient, who had prior caregiving experience, and who also needed to care for children exhibited higher levels of supportive care needs (*p* < 0.05).

**Conclusions:**

Our findings indicate that family caregivers of patients with multiple myeloma have significant unmet needs and emphasize the necessity for more investment and resources in this area.

**Supplementary Information:**

The online version contains supplementary material available at https://doi.org/10.1186/s12904-026-02312-9.

## Background

Multiple myeloma (MM) is a malignant tumor that occurs in incompletely differentiated plasma cells and is characterized by an uncontrolled increase in monoclonal paraproteins, eventually leading to tissue or organ damage [1]. MM is an incurable disease with high morbidity and mortality rates, particularly in older adults. According to the latest Global Cancer (GLOBOCAN) statistics, there are approximately 176,000 cases of MM worldwide [2]. MM is the second most common hematological malignancy after leukemia [3]. With the development of new medications, cellular immunotherapy, and hematopoietic stem cell transplantation technology, the survival period of patients with MM has been significantly extended [4]. However, the adverse reactions caused by the disease and its treatment have severely affected patients’ quality of life [5].

The treatment of MM is a lengthy and intricate process, requiring not only professional support but also physical, psychological, and social assistance from family caregivers (FCs) [6]. FCs, including parents, children, and spouses, devote substantial time and energy to providing unpaid care to patients [7]. As treatment for MM is increasingly administered in outpatient clinics, FCs play a vital role in providing extensive practical and emotional support for patients [8]. However, unexpected caregiving responsibilities may put patients’ health at risk [9]. Research shows that most caregivers lack the specialized training necessary to ensure quality care and are not emotionally prepared to assume the caregiver role, potentially leading to an increased burden [10, 11]. Caregivers often experience higher levels of psychological distress and a lower quality of life than patients [9, 12, 13]. A survey showed that nearly half (44.4%) of caregivers of patients with MM reported significant levels of anxiety [14].

Studies have indicated a substantial need for supportive care among caregivers, which includes assistance beyond treatment, spanning from the time of cancer diagnosis to advanced stages or cure [15, 16]. It is necessary to increase supportive care supply for caregivers; such as the support services, information, and resources related to patient care. When the supportive care supply is less than the need, caregivers’ needs are unmet. The ability of caregivers to cope with disease may be compromised [16], which increases their psychological distress [17–19] and even leads to physical symptoms such as headache, insomnia, and gastrointestinal reactions [20]. In a systematic review of cancer caregivers’ needs, almost half of the studies reported that over 20% of caregivers had unmet needs [21]. FCs of patients with MM experience a heavier task burden, lower quality of life, and require more support from health professionals than caregivers of patients with other types of cancer [22].

Despite the significant burden of the disease on FCs of patients with MM, FC’s supportive care needs and the perceived supply remain poorly understood [12, 23]. Qualitative studies have been conducted to identify the needs of FCs of patients with MM; however, the general needs of most caregivers cannot be fully understood because of research limitations [23, 24]. Molassiotis et al. used the Cancer Survivors’ Partners Unmet Needs Measure (CaSPUN) to investigate the supportive care needs of their partners who cared for patients with MM and found that one-third of the partners reported unmet needs [12]. However, limitations persist because of the lack of precise tools available to measure the specific needs of FCs of patients with MM.

Given that, our study used a tailored questionnaire to evaluate the supportive care needs and perceived supply in FCs of patients with MM. We aimed to provide a basis for medical staff to meet caregivers’ supportive care needs by conducting a comprehensive analysis of the unmet needs and identifying associated factors.

## Methods

### Study design and participants

This was a cross-sectional study. Participants were recruited from January 2021 to December 2021 in the outpatient and inpatient departments of hematology at a large tertiary hospital in Fujian Province, China. It should be noted that although our study was conducted during the COVID-19 pandemic, this outbreak was greatly under control in the city of Fuzhou and did not significantly affect our study. The patients and caregivers were able to receive offline consultations and obtain support from healthcare professionals, using nucleic acid test reports as a guide. Also, as usual, we provided telephone numbers of the wards to facilitate the provision of online support and services.

Convenience sampling was used to select participants. Caregiver inclusion criteria were as follows: (1) age ≥ 18 years; (2) FCs of patients diagnosed with MM, as documented in medical records, including spouses, parents, children, and siblings; (3) assuming primary caregiving responsibilities; and (4) knowledge of the patient’s disease diagnosis, condition, and treatment. Caregivers with severe physical or mental illnesses were excluded. A priori power analysis was performed using G Power 3.1 [25]. A linear multiple regression test was used with α = 0.05 (two tails), an effect size of d = 0.15, and a power (1-β) of 0.90 to compute the required sample size, which was 198. This study adhered to the Strengthening the Reporting of Observational Studies in Epidemiology checklist [26].

### Measurements

Participants completed a questionnaire regarding their sociodemographic characteristics (such as age, sex, and employment), their supportive care needs, and their supportive care perceived supply. Patient characteristics were directly extracted from the patients’ medical records with consent.

#### Sociodemographic characteristics

For caregivers, sociodemographic information included age, sex, educational level, marital status, employment, monthly consumption per capita, health status, relationship with the patient, caregiving experience, and daily caregiving time.

#### Questionnaire on supportive care needs and perceived supply among FCs of patients with MM

This questionnaire was used to assess supportive care needs and perceived supply among FCs of patients with MM. Developed by our research team, it was based on the published Cancer Patient Caregiver Needs Scale and combined with semi-structured interviews and three rounds of Delphi expert consultations [27]. The questionnaire consisted of two parts, needs and perceived supply, both of which had identical items and were tested before application. These two parts each contained 29 items covering four domains: disease information (10 items), caregiving skills (10 items), psychosocial resources (4 items), and medical services (5 items). All items were rated on a 5-point Likert scale ranging from 1 (not needed or not perceived supply) to 5 (greatly needed or high level of perceived supply), with higher scores indicating greater need or higher perceived supply of supportive care.

The total content validity index (CVI) for the questionnaire was 0.998, with item CVIs between 0.93 and 1.00. The needs part had a Cronbach’s α of 0.962, split-half reliability of 0.845, and retest reliability of 0.883. For the part on perceived supply, Cronbach’s α was 0.981, split-half reliability was 0.914, and retest reliability was 0.802. The exploratory factor analysis of the needs part extracted four metrics with a cumulative variance contribution of 70.160%, while the part on perceived supply was based on the needs part, and only one metric factor was extracted for each dimension, with cumulative variance contributions of 73.803%, 80.528%, 80.486%, and 74.666%. Validation factor analysis indicated good model fit and stable factor structure for the two parts.

### Data collection

Data collection was conducted by a researcher at the hospital. The purpose, content, and procedures of the study were explained to eligible caregivers prior to their participation. In particular, it needs to be fully stated that the needs part of the questionnaire referred to the participant’s most desired support in the care of the patient, and the part on perceived supply referred to the participant’s perception of support they had received from healthcare professionals. The participants completed all questionnaires themselves. For those who had difficulty completing the questionnaire, the research assistant read the questions and options without inducing responses. Upon completion, the questionnaires were immediately checked for any omissions. If omissions were detected, participants were instructed to complete them.

### Statistical analyses

The collected questionnaires were uniformly coded and checked by two investigators before data entry. Data entry and statistical analyses were performed using SPSS software (version 24.0; IBM Corp., Armonk, NY, USA). Descriptive statistics were used to report the participants’ characteristics, supportive care needs, and perceived supply. Participants’ characteristics were described using frequencies and percentages, while supportive care needs and perceived supply were described using means with standard deviations. A paired-sample t-test was used to compare the gap between the needs and perceived supply of FCs of patients with MM and focused on the top 10 unmet needs to address the major problems currently faced by caregivers. Variables (*p* < 0.05) in the independent samples t-test (two groups) and one-way analysis of variance (ANOVA) (multiple groups) were put into multiple linear regression to further analyze the factors influencing supportive care needs. All tests were two-tailed, and a p-value of less than 0.05 was considered statistically significant.

### Ethical considerations

This study was approved by the Ethics Committee of our institution. All participants read and signed informed consent forms. They were informed that participation was voluntary and that any identifying information would be anonymized and protected. All methods were conducted according to the ethical principles stated in the Declaration of Helsinki.

## Results

### Participants’ characteristics

Two hundred and fifty-one FCs of patients with MM were approached, of which 24 caregivers declined to participate owing to lack of interest in research (*n* = 13), time constraints (*n* = 7), or unspecified reasons (*n* = 4). Ultimately, 227 caregivers completed this survey. Among them, 52.4% were under 50 years of age, 54.6% were female, and 50.7% were spouses of the patient. Most caregivers (77.1%) dedicated more than 6 h per day to patient care. Only 11.0% had previous caregiving experience, 47.6% provided care without assistance from other caregivers, 28.6% needed to care for others, and 15.9% had chronic diseases. The characteristics of both caregivers and patients are described in Table 1.

**Table 1:** Socio-demographic and clinical characteristics, and the associations with the supportive care needs (*n* = 227)

Variables	*n* (%)	Supportive care needs score (Mean ± SD)	F/t	*P*
Caregivers				
Age, year			0.870^a^	0.457
≤ 50	119 (52.4)	124.65 ± 17.40		
51 ~ 60	65 (28.6)	123.11 ± 15.86		
61 ~ 70	33 (14.6)	119.95 ± 15.78		
≥ 71	10 (4.4)	120.20 ± 15.99		
Gender			-0.443^b^	0.658
Female	124 (54.6)	122.71 ± 15.27		
Male	103 (45.4)	123.69 ± 17.75		
Education level			0.608^a^	0.610
Primary or below	46 (20.3)	124.33 ± 18.02		
Junior high school	80 (35.2)	121.73 ± 14.68		
Senior high school or technical secondary school	43 (18.9)	122.26 ± 15.10		
College or above	58 (25.6)	125.22 ± 19.14		
Marital status			-0.393^b^	0.695
Married	201 (88.6)	123.40 ± 16.16		
Others	26 (11.4)	122.04 ± 20.34		
Employment			0.491^a^	0.613
Employed	66 (29.1)	124.85 ± 19.96		
Part-time/ Freelance	100 (44.0)	122.95 ± 14.63		
Retired	61 (26.9)	122.00 ± 15.92		
Monthly consumption per capita, RMB			0.955^a^	0.386
< 1000	26 (11.5)	127.46 ± 13.29		
1000 ~ 3000	165 (72.7)	122.61 ± 16.72		
3001 ~ 5000	36 (15.8)	123.11 ± 18.37		
Place of residence			0.518^a^	0.596
City	107 (47.1)	123.74 ± 17.91		
Townships	55 (24.2)	121.27 ± 16.22		
Countryside	65 (28.7)	124.11 ± 14.84		
Health status			-1.090^b^	0.280
Good	191 (84.1)	122.82 ± 17.25		
Suffering from chronic diseases	36 (15.9)	125.53 ± 12.90		
Relationship with patients			1.276^a^	0.281
Spouse	115 (50.7)	124.29 ± 14.12		
Children	97 (42.7)	122.97 ± 17.65		
Others	15 (6.6)	117.07 ± 25.68		
Religion			-1.860^b^	0.064
No	182 (80.2)	122.23 ± 16.62		
Yes	45 (19.8)	127.36 ± 16.26		
Knowledge of the disease			0.513^a^	0.600
Not at all	3 (1.3)	114.67 ± 8.08		
A little	147 (64.8)	123.74 ± 16.21		
A lot	77 (33.9)	122.63 ± 17.71		
Care experience			-3.944^b^	< 0.001
No	202 (89.0)	122.13 ± 16.87		
Yes	25 (11.0)	132.24 ± 11.36		
Daily caregiving time, hour			0.549^a^	0.578
< 6	52 (22.9)	125.37 ± 19.94		
6 ~ 12	85 (37.5)	122.74 ± 17.46		
> 12	90 (39.6)	122.50 ± 13.57		
Assistance from other caregivers			0.322^b^	0.748
No	108 (47.6)	123.62 ± 16.31		
Yes	119 (52.4)	122.91 ± 17.00		
Need to care for others			4.451^a^	0.013
No	162 (71.4)	121.45 ± 17.00		
Children	35 (15.4)	130.46 ± 15.61		
Others	30 (13.2)	124.53 ± 13.60		
Patients				
Age, year			0.202^a^	0.817
< 60	105 (46.3)	123.03 ± 16.94		
60 ~ 69	74 (32.6)	124.18 ± 15.66		
≥ 70	48(21.1)	122.29 ± 17.71		
Gender			0.233^b^	0.816
Female	97 (42.7)	122.95 ± 15.42		
Male	130 (57.3)	123.47 ± 17.55		
Disease duration			1.054^a^	0.370
< 3 months	71 (31.3)	125.39 ± 17.16		
3–6 months	38 (16.7)	124.08 ± 14.88		
6–12 months	17 (7.5)	125.12 ± 15.66		
> 12 months	101 (44.5)	121.11 ± 17.02		
Treatment stage			3.015^a^	0.051
Initial treatment	72 (31.7)	125.67 ± 17.20		
Consolidation therapy	112 (49.3)	123.71 ± 16.61		
Relapse treatment	43 (19.0)	117.98 ± 14.90		
Type of medical insurance			5.591^a^	0.004
Self-funded or otherwise	17 (7.5)	124.71 ± 20.16		
Rural medical insurance	87 (38.3)	127.57 ± 15.85		
Urban medical insurance	123 (54.2)	119.98 ± 16.06		
Whether the treatment hospital is in the residence			-0.766^b^	0.446
No	161 (70.9)	123.84 ± 15.35		
Yes	66 (29.1)	121.79 ± 19.48		

^a^=F, ^b^=t

### Supportive care needs and perceived supply among FCs of patients with MM

The overall score for the supportive care needs among FCs of patients with MM was (123.25 ± 16.64), with the ranking order of scores in each domain as follows: disease information > medical services > caregiving skills > psychosocial resources. The total score for the supportive care perceived supply among FCs of patients with MM was (91.61 ± 23.24), with the ranking order of scores in each domain as follows: disease information > caregiving skills > medical services > psychosocial resources (Table 2).

**Table 2:** The supportive care needs and percrived supply of MM patients’ FCs (-x ± s)

Domains	Score range	Needs (Mean ± SD)	Needs rate(%)	Perceived supply (Mean ± SD)	Perceived supply rate(%)
Total score	29 ~ 145	123.25 ± 16.64	85.00	91.61 ± 23.24	63.18
Disease information	10 ~ 50	45.02 ± 5.15	90.04	35.47 ± 8.48	70.94
Care skills	10 ~ 50	41.38 ± 7.02	82.76	30.91 ± 9.79	61.82
Psychosocial resources	4 ~ 20	15.42 ± 3.63	77.10	10.01 ± 3.90	50.05
Medical services	5 ~ 25	21.42 ± 3.35	85.68	15.22 ± 4.78	60.88

Needs rate = Total score / Number of items * Full score of items

Perceived supply rate = Total score / Number of items * Full score of items

A substantial disparity existed between the needs and perceived supply across all domains and item scores (*p*<0.001). Among the top 10 unmet needs with the biggest disparity between need and perceived supply, four items were for psychosocial resources, three items for caregiving skills, two items for medical services, and one item for disease information (Table 3).

**Table 3:** The needs-perceived supply gap of MM patients’ FCs (-x ± s)

Items	Needs	Perceived supply	Needs-perceived supply gap	t	*P*	Needs-perceived supply gap ranking
Disease information	45.02 ± 5.15	35.47 ± 8.48	9.55 ± 9.32	15.441	< 0.001	
D9: Provide knowledge about protecting kidney function	4.41 ± 0.70	3.24 ± 1.10	1.17 ± 1.22	14.516	< 0.001	10
D5: Informs you of the results about physical examination and guides you in recognizing abnormalities and risk values	4.55 ± 0.65	3.39 ± 1.12	1.15 ± 1.29	13.453	< 0.001	13
D6: Inform you of the effects, use method, precautions and common adverse reactions of the drug	4.62 ± 0.56	3.53 ± 1.09	1.09 ± 1.19	13.853	< 0.001	16
D4: Assist you in obtaining information about patient’s current disease status, future course of treatment and prognosis	4.56 ± 0.59	3.56 ± 1.05	1.01 ± 1.15	13.193	< 0.001	18
D3: Inform you of knowledge related to the examination of myeloma	4.40 ± 0.63	3.40 ± 1.01	1.00 ± 1.08	13.853	< 0.001	19
D8: Inform you of the patient’s dietary precautions during hospitalization and after discharge	4.55 ± 0.61	3.58 ± 1.12	0.97 ± 1.24	11.858	< 0.001	21
D2: Introduces you to the main treatment programs and efficacy both domestically and internationally	4.42 ± 0.73	3.57 ± 1.06	0.85 ± 1.20	10.684	< 0.001	24
D7: Inform you of the patient’s safe activities during hospitalization and after discharge	4.57 ± 0.56	3.72 ± 1.00	0.85 ± 1.04	12.438	< 0.001	25
D10: Provide you with the process and scope of medical insurance reimbursement	4.50 ± 0.68	3.68 ± 1.06	0.82 ± 1.17	10.548	< 0.001	27
D1: Introduce you to the knowledge of multiple myeloma disease	4.43 ± 0.77	3.80 ± 0.92	0.63 ± 1.11	8.513	< 0.001	29
Care skills	41.38 ± 7.02	30.91 ± 9.79	10.47 ± 10.80	14.614	< 0.001	
C10: Instruct you on ways to assist patients with pain relief	4.33 ± 0.75	3.03 ± 1.06	1.31 ± 1.31	14.998	< 0.001	6
C4: Instruct you to prevent and observe whether blood clots	4.20 ± 0.81	2.95 ± 1.24	1.25 ± 1.36	13.776	< 0.001	7
C3: Instruct you on ways to prevent, observe, and manage bleeding	4.12 ± 0.94	2.95 ± 1.25	1.18 ± 1.31	13.508	< 0.001	9
C6: Instructs you on ways to prevent, observe, and manage lung infections	4.32 ± 0.80	3.16 ± 1.17	1.16 ± 1.28	13.677	< 0.001	11
C5: Instruct you on ways to prevent, observe, and manage oral infections	4.21 ± 0.86	3.11 ± 1.22	1.10 ± 1.39	11.873	< 0.001	14
C8: Provides guidance on maintaining the clarity of the patient’s bowel movements	4.21 ± 0.82	3.12 ± 1.17	1.09 ± 1.27	12.878	< 0.001	15
C1: Instructs you on ways to assist the patients in turning over	4.25 ± 0.88	3.28 ± 1.17	0.97 ± 1.37	10.676	< 0.001	20
C9: Instruct you on ways to assist patients with exercise and related precautions	4.31 ± 0.77	3.36 ± 1.07	0.96 ± 1.12	12.904	< 0.001	22
C7: Instruct you on ways to prevent, observe, and manage perianal infections	4.07 ± 0.96	3.24 ± 1.17	0.83 ± 1.34	9.363	< 0.001	26
C2: Instruct you on routine maintenance of central venous catheters	3.35 ± 1.47	2.72 ± 1.46	0.63 ± 1.48	6.447	< 0.001	28
Psychosocial resources	15.42 ± 3.63	10.01 ± 3.91	5.41 ± 4.70	17.354	< 0.001	
P3: Give you advice on raising treatment funds	3.68 ± 1.18	2.16 ± 1.18	1.52 ± 1.52	15.086	< 0.001	2
P4: Provides you with the opportunity to communicate with other experienced caregivers	3.96 ± 0.97	2.58 ± 1.25	1.38 ± 1.41	14.775	< 0.001	3
P1: Guidance on cope with negative emotions such as anxiety, depression, nervousness or irritability	3.95 ± 1.07	2.63 ± 1.09	1.32 ± 1.32	15.008	< 0.001	5
P2: Provides you with the skills to communicate with patients	3.82 ± 1.09	2.63 ± 1.05	1.19 ± 1.29	13.878	< 0.001	8
Medical services	21.42 ± 3.35	15.22 ± 4.78	6.20 ± 4.97	18.81	< 0.001	
M2: Set up a self-learning online course on multiple myeloma health education	4.05 ± 0.91	2.30 ± 1.28	1.75 ± 1.42	18.618	< 0.001	1
M1: Conducting health education activities	4.11 ± 0.93	2.75 ± 1.23	1.36 ± 1.33	15.326	< 0.001	4
M5: Medical staff provides online answers to patient questions	4.48 ± 0.70	3.33 ± 1.34	1.15 ± 1.40	12.337	< 0.001	12
M4: Provide you with contact information for medical and nursing team	4.48 ± 0.71	3.40 ± 1.26	1.08 ± 1.29	12.702	< 0.001	17
M3: The hospital can contact you to follow up with the patient	4.30 ± 0.72	3.44 ± 1.09	0.87 ± 1.17	11.2	< 0.001	23

D1-D10 = Disease information, C1-C10 = Care skills

P1-P4 = Psychosocial resources, M1-M5 = Medical services

The top five supportive care needs were related to disease information, mainly focusing on guidance concerning medication, activities, diet, examination results, and other disease-related information. In the bottom five supportive care perceived supply items, four were for psychosocial resources and one for medical services. These included communication skills with patients, coping strategies for negative emotions, opportunities to interact with other caregivers, online health education courses, and advice on raising treatment funds (Table 4).

**Table 4:** The top 5 and bottom 5 of supportive care needs and perceived supply of MM patients’ FCs

	Domains	Items	Mean ± SD
The top 5 supportive care needs	Disease information	1. Inform you of the effects, use method, precautions and common adverse reactions of the drug	4.62 ± 0.56
		2. Inform you of the patient’s safe activities during hospitalization and after discharge	4.57 ± 0.56
		3. Assist you in obtaining information about patient’s current disease status, future course of treatment and prognosis	4.56 ± 0.59
		4. Inform you of the patient’s dietary precautions during hospitalization and after discharge	4.55 ± 0.61
		5. Informs you of the results about physical examination and guides you in recognizing abnormalities and risk values	4.54 ± 0.65
The bottom 5 supportive care perceived supply	Psychosocial resources	1. Provides you with the skills to communicate with patients	2.63 ± 1.05
		2. Guidance on cope with negative emotions such as anxiety, depression, nervousness or irritability	2.63 ± 1.09
		3. Provides you with the opportunity to communicate with other experienced caregivers	2.58 ± 1.25
		4. Give you advice on raising treatment funds	2.16 ± 1.18
	Medical services	5. Set up a self-learning online course on multiple myeloma health education	2.30 ± 1.28

### Factors associated with supportive care needs of FCs of patients with MM

Univariate analysis revealed that care experience, need to care for others, and medical insurance were factors influencing the supportive care needs of FCs of patients with MM (Table 1).

With supportive care needs as the dependent variable, factors with statistical significance from the univariate analysis, along with variables that were correlated with supportive care needs, were used as independent variables for multiple linear regression analysis (Table 5). The results suggested that patients with rural medical insurance, patients in the primary treatment stage or consolidation therapy stage, caregivers who were spouses of the patients, those with previous caregiving experience, and those who need to care for children were significantly correlated with the supportive care needs (R^2^ = 0.207, F = 2.690, *P* < 0.001) (Table 6).

**Table 5:** Variable assignment methods

Variables	Assignment method
Caregivers’ age, year	≤ 50 = 0, 51 ~ 60=1, 61 ~ 70=2, ≥ 71 = 3
Education level	primary or below = 0, junior high school = 1, senior high school or technical secondary school = 2, college or above = 3
Marital status	married = 0, others = 1
Health status	good = 0, suffering from chronic diseases = 1
Relationship with patients	Setting dummy variables with reference to “others” X1: spouse = 1, children = 0, others = 0 X2: children = 1, spouse = 0, others = 0
Religion	no = 0, yes = 1
Knowledge of the disease	not at all = 0, a little = 1, a lot = 2
Care experience	no = 0, yes = 1
Assistance from other caregivers	no = 0, yes = 1
Need to care for others	Setting dummy variables with reference to “no” X1: children = 1, no = 0, others = 0 X2: others = 1, no = 0, children = 0
Treatment stage	Setting dummy variables with reference to “relapse treatment” X1: initial treatment = 1, consolidation therapy = 0, relapse treatment = 0 X2: consolidation therapy = 1, initial treatment = 0, relapse treatment = 0
Type of medical insurance	Setting dummy variables with reference to “urban medical insurance” X1: self-funded or otherwise = 1, rural medical insurance = 0, urban medical insurance = 0 X2: rural medical insurance = 1, self-funded or otherwise = 0, urban medical insurance = 0
Whether the treatment hospital is in the residence	no = 0, yes = 1

**Table 6:** Factors associated with supportive care needs of MM patients’ FCs

Variables		B(SE)	95% CI	*P*
Type of medical insurance (rural medical insurance)	5.660(2.409)		0.910 ;10.411	0.020
Treatment stage (primary treatment)	6.800(3.205)		0.481 ;13.118	0.035
Treatment stage (consolidation therapy)	7.411(2.920)		1.653 ;13.169	0.012
Relationship with patients(spouse)	15.142(4.858)		5.563 ;24.720	0.002
Care experience (have care experience)	9.023(3.580)		1.964 ;16.082	0.012
Need to care for others (children)	11.182(3.254)		4.766 ;17.597	0.001

R^2^ = 0.207, R^2^ adjusted = 0.130, F = 2.690, *P* < 0.001

## Discussion

This study provides insights into the current supportive care needs and perceived supply among FCs of patients with MM. A significant imbalance exists between supply and demand, and a high level of unmet needs persists. Notably, more investment and resources are required in this area in the future.

Unmet needs span all domains, with psychosocial resources being the most prominent among the top 10 unmet needs, which is consistent with the findings of previous studies [28, 29]. Our study showed that caregivers’ need for psychosocial resources ranked last, potentially resulting in decreased psychosocial services provided by medical personnel. Nevertheless, that does not mean caregivers do not need psychosocial services. The caregivers often overlooked or even concealed their own psychosocial needs due to concentrating on patient caregiving. A qualitative study by Quinoa-Salanova et al. identified that caregivers hide their inner needs to avoid worrying others [23]. This was also confirmed in a study by Morris et al. on the experience of MM patients’ caregivers during home-based oral chemotherapy treatment [30]. This suggests that the psychosocial needs of caregivers cannot be ignored and educational interventions or emotional management programs are necessary to meet their psychological needs [31]. Additionally, knowledge and skills of pain relief, thrombosis management, and renal function protection should be prioritized. Impairment of renal function, bone disease, and thromboembolic events are highly prevalent in patients with MM, necessitating caregivers to enhance their capacity in managing these symptoms [32]. Implementing health education activities and setting up self-learning through MM health education courses online can address the various needs of caregivers for disease information and caregiving skills [33, 34].

Caregivers have the highest supportive care needs for disease information, but only one of the top 10 unmet needs belonged to this area, indicating that health care workers have invested more effort in this area. It should be noted that the needs related to patient safety activities ranked second among the top five supportive care needs. This result indicates that there is a need to increase information about patient safety activities for caregivers. Cancer increases the risk of pathological fractures [35, 36], particularly in patients with MM, with over 80% experiencing pathological fractures during disease progression [37]. Due to the bone destruction by myeloma cells, bone calcium loss, tumor infiltration, and medication side effects, MM patients are susceptible to fracture from the slightest external force in their daily activities. Health education manuals [38], classroom instruction courses, educational videos [39], and video conferencing [40] can be used to raise the awareness of safety activities for caregivers and patients.

Factors associated with the supportive care needs of FCs of patients with MM include sociodemographic and clinical characteristics. We found that the needs of caregivers were higher when patients were enrolled in rural medical insurance. Rural medical insurance reimburses less than urban health insurance, meaning higher out-of-pocket medical costs. In addition, there are fewer employment opportunities and unstable incomes in rural areas. All of these factors lead to higher financial burdens on their families. This result supports the findings that economics is a significant predictor of the supportive care needs of FCs [41, 42]. Cancer not only increases financial expenditure but may also hinder caregivers from maintaining their work routines, exacerbating the loss of income and welfare of the family [43, 44]. The needs of caregivers were higher when patients were in the primary or consolidation treatment phases, which aligns with the results of a previous study [16]. During the initial treatment stage, caregivers with limited experience may require additional support to manage the disease. Over time, their supportive care needs will decrease as they accumulate disease knowledge, nursing skills, and experience to reduce care burden [45]. Therefore, it is crucial to assess regularly and provide adequate support to caregivers when patients are in the initial treatment stage.

Our research indicates that spouses had higher demands, which is consistent with the findings of Sakiko et al. [46]. On the one hand, spouses experience higher levels of anxiety and burden than other family members [47, 48]. On the other hand, influenced by traditional Chinese concepts, spouses usually regard caregiving duties as an obligation in marriage [49]. Caregivers with more caregiving experience had greater needs than those with less or no experience. Previous caregiving experience had made them aware of their shortcomings in caring for patients, so they reported more needs to compensate for perceived inadequacies. FCs who needed to care for children in addition to the patient also had higher supportive care needs. As they are not just a caregiver, they are also a parent, a child, an employee, and so on. The multiple roles assumed by a single individual substantially increase their burden [50]. Being responsible for many individuals demands considerable energy and time, and the heavy burden of providing long-term care can affect the caregivers’ health [11]. Social support from friends and other family members can lessen the burden on caregivers and improve their overall well-being [51, 52]. In our study, the effects of caregivers’ age and education level on supportive care needs were not statistically significant, which differs from the findings of Zhang et al. [53]. This might be because of the limited sample size, and the statistically significant variables may have masked their influences. The effects of both age and education level require further study.

### Limitations

This study had a few limitations. First, as it was a cross-sectional study, prospective research is required to validate our results. Second, it analyzed the FCs of patients with MM in the hematology department of only one hospital; therefore, caution is required when generalizing the results to caregivers in different regions. Future studies should expand the sample size and include multiple centers. Third, because convenience sampling was used, the possibility of bias in participant selection must be considered. Fourth, the cross-sectional design did not capture longitudinal changes in caregivers’ needs; this could be addressed by conducting longitudinal studies in the future studies.

## Conclusion

Our study provides an understanding of the supportive care needs and perceived supply among FCs of patients with MM and extends the current research in this area. Unmet supportive care needs are common among FCs of patients with MM, and certain needs require particular attention. Medical staff should focus on caregivers’ psychosocial resources and caregiving skills. Specifically, caregivers whose patients have rural medical insurance, those with patients in the primary or consolidation treatment stages, those who are spouses of the patients, those with previous caregiving experience, and those who also need to care for children require more attention.

## Supplementary Information

Supplementary Material 1.

## Data Availability

The datasets used and/or analyzed during the current study are available from the corresponding author on reasonable request.

## Abbreviations

MM: Multiple myeloma
GLOBOCAN: Global cancer
FCs: Family caregivers
CaSPUN: Cancer Survivors’ Partners Unmet Needs
CVI: Content Validity Index
