# Supplementary material for: The unbalanced needs and perceived supply of supportive care in family caregivers of patients with multiple myeloma: a cross-sectional study
Source: BMC Palliat Care. 2026 Aug 29;25:321. doi: 10.1186/s12904-026-02312-9 (PMC13693206; doi:10.1186/s12904-026-02312-9)
Supplement: Supplementary file 1 [file 12904_2026_2312_MOESM1_ESM.docx]

**Delphi** **Expert Consultation of the Questionnaire on Supportive Care Needs and Perceived Supply among Family Caregivers of Patients with Multiple Myeloma**

1.Method

1.1Expert selection

Our study invited 15 specialists: 6 hematology medical specialists and 9 hematology nursing specialists, all of them over 40 years old. Among them, 73.3% of specialists worked for more than 20 years. Seven specialists held senior titles, and eight specialists had associate senior titles. Sixty percent of specialists held master's or doctoral degrees, with 6 specialists being master's supervisors and 4 specialists being doctoral supervisors.

1.2 Consultation material

There are three parts. The first part described the purpose and the method of this consultation. The second part is the content of this consultation. Experts are required to make a judgment on its importance of each item, using a 5-point Likert scale, very important = 5 points, important = 4 points, average = 3 points, unimportant = 2 points, and very unimportant = 1 point. The third part is the survey of experts' situations, including the information of experts, the degree of familiarity of the expert with the consultation material, and the basis of judgment. Consultation materials were sent and returned by e-mail. In order to avoid the bias of memory, the interval between pre- and post-consultation was more than 20 days, and the period of each round of consultation was 1–2 weeks.

1.3 Statistical indicators

1.3.1 Degree of expert positivity

This is the degree of experts' concern for this study. The higher the degree of expert positivity, the higher the credibility of the feedback results. The expert positivity coefficient was evaluated by the effectiveness of questionnaire recovery and the rate of experts' suggestions. The recovery rate >50% to be included.

1.3.2 Degree of expert authority

It is determined by the judgment basis of expert and the degree of familiarity of the expert with the investigation content. There are four parts of the expert judgment basis, including practical experience, theoretical basis, reference to domestic and international literature, and intuition. Experts evaluate themselves according to three levels of influence: large, medium, and small. The degree of content familiarity is self-assessed by experts according to five levels: very familiar, relatively familiar, generally familiar, not too familiar, and unfamiliar. The researcher assigns values to the expert's influence and their familiarity with the content according to the formula: authority coefficient (Cr) = (basis of judgment (Ca) + degree of familiarity (Cs)) / 2. An expert authority coefficient >0.7 is generally considered to be acceptable.

1.3.3 Concentration of expert opinion

Two aspects were evaluated, including the mean ± standard deviation of the entries and the coefficient of variation. It is generally accepted that the larger the mean and the smaller the standard deviation, the higher the importance of the entry. The coefficient of variation reflects the fluctuation of each expert's evaluation of the importance of an entry. The smaller the coefficient of variation, the more concentrated the experts' opinions are. In our study, the criteria for selection of entries were set as follows: mean importance rating >4.0 and coefficient of variation <0.20.

1.3.4 Degree of coordination of expert opinions

Kendall's coordination coefficient (Kendall's W) is used, which is between 0 and 1, the larger the score indicates the higher the degree of coordination of experts.

2.Result

2.1 Degree of expert positivity

In this study, 15 consultation materials were sent in the first two rounds and all were recovered. In the third round, 14 consultation materials were sent and all were recovered. All the consultation materials were valid, and the validity rate was 100%. The percentage of experts' opinions was 73.3%, 20%, and 14.3%, respectively, which indicates that the degree of expert positivity was high.

2.2 Degree of expert authority

In the three rounds of expert consultation, Cr was above 0.80. Table 1 and Table 2 for details.

Table 1 Statistics of authority coefficients of experts in the first and second rounds

| Expert number | Ca | Cs | Cr |
| --- | --- | --- | --- |
| 1 | 1.00 | 1.00 | 1.00 |
| 2 | 1.00 | 1.00 | 1.00 |
| 3 | 1.00 | 1.00 | 1.00 |
| 4 | 1.00 | 1.00 | 1.00 |
| 5 | 1.00 | 0.80 | 0.90 |
| 6 | 1.00 | 1.00 | 1.00 |
| 7 | 1.00 | 0.80 | 0.90 |
| 8 | 0.80 | 0.80 | 0.80 |
| 9 | 1.00 | 1.00 | 1.00 |
| 10 | 0.90 | 1.00 | 0.95 |
| 11 | 0.80 | 1.00 | 0.90 |
| 12 | 1.00 | 1.00 | 1.00 |
| 13 | 0.90 | 0.80 | 0.85 |
| 14 | 0.90 | 0.80 | 0.85 |
| 15 | 1.00 | 1.00 | 1.00 |
| Mean | 0.95 | 0.93 | 0.94 |

Note: Ca= basis of judgment, Cs= degree of familiarity, Cr= coefficient of authority

Table 2 Statistics of the third round of expert authority coefficient

| Expert number | Ca | Cs | Cr |
| --- | --- | --- | --- |
| 1 | 1.00 | 1.00 | 1.00 |
| 2 | 1.00 | 1.00 | 1.00 |
| 3 | 1.00 | 1.00 | 1.00 |
| 4 | 1.00 | 1.00 | 1.00 |
| 5 | 1.00 | 0.80 | 0.90 |
| 6 | 1.00 | 1.00 | 1.00 |
| 7 | 1.00 | 0.80 | 0.90 |
| 8 | 0.80 | 0.80 | 0.80 |
| 9 | 1.00 | 1.00 | 1.00 |
| 10 | 0.90 | 1.00 | 0.95 |
| 11 | 0.80 | 1.00 | 0.90 |
| 12 | 1.00 | 1.00 | 1.00 |
| 13 | 0.90 | 0.80 | 0.85 |
| 14 | 1.00 | 1.00 | 1.00 |
| Mean | 0.96 | 0.94 | 0.95 |

Note: Ca= basis of judgment, Cs= degree of familiarity, Cr= coefficient of authority

2.3 Concentration of expert opinion

The first round of consultation showed that the mean of the importance of the dimensions of the questionnaire ranged from 4.33 to 4.87, the mean of the importance of the entries ranged from 3.20 to 4.80, the coefficient of variation (CV) of the dimensions ranged from 0.07 to 0.14, and the CV of the entries ranged from 0.09 to 0.30.

The second round of consultation showed that the mean of the importance of the dimensions of the questionnaire ranged from 4.40 to 4.93, the mean of the importance of the entries ranged from 3.80 to 5.00, the coefficient of variation (CV) of the dimensions ranged from 0.05 to 0.14, and the CV of the entries ranged from 0.00 to 0.22.

The third round of consultation showed that the mean of the importance of the dimensions of the questionnaire ranged from 4.50 to 5.00, the mean of the importance of the entries ranged from 4.00 to 5.00, the coefficient of variation (CV) of the dimensions ranged from 0.00 to 0.12, and the CV of the entries ranged from 0.00 to 0.15. After three rounds of consultation, there was a convergence of expert opinion.

2.4 Degree of coordination of expert opinions

In this study, the third round of expert consultation showed a Kendall's W of 0.412 for dimensions and 0.402 for entries, indicating a high degree of coordination of expert opinions. For details, Table 3.

Table 3 Kendall's W and its significance test results of three rounds of expert opinions

| Times |  | Number of entries | Kendall’s W |  | df | *P* |
| --- | --- | --- | --- | --- | --- | --- |
| First round | dimensions | 4 | 0.364 | 16.375 | 3 | 0.001 |
|  | entries | 32 | 0.352 | 163.567 | 31 | <0.001 |
| Second round | dimensions | 4 | 0.342 | 15.393 | 3 | 0.002 |
|  | entries | 32 | 0.248 | 115.263 | 31 | <0.001 |
| Third  round | dimensions | 4 | 0.412 | 17.294 | 3 | 0.001 |
|  | entries | 29 | 0.402 | 157.575 | 28 | <0.001 |

2.5 Revision of questionnaire entries

We revised some entries according to the opinions of the experts. C2, C3, C5, and D6 were deleted. D2 and D3 were merged, and A4, A6, D1, and D5 were modified to improve comprehension, clarity, and relevance. D4 was changed to “Provide accurate contact information of the medical and nursing team, to facilitate timely contact with medical and nursing staff when necessary.” Following three rounds of expert consultation, the initial draft of the Questionnaire on Supportive Care Needs and Perceived Supply of Family Caregivers of Patients with Multiple Myeloma was developed, comprising 29 items across four dimensions.

**Questionnaire on Supportive Care Needs and Perceived Supply among Family Caregivers of Patients with Multiple Myeloma (Initial draft)**

Please fill in according to your actual situation, and tick "√" in the box that you think is most suitable.

|  |  | Needs (you need it) | | | | | Supply (you got it) | | | | |
| --- | --- | --- | --- | --- | --- | --- | --- | --- | --- | --- | --- |
|  |  | Very much | Quite a bit | Medi-um | A little bit | Not at all | Very much | Quite a bit | Medi-um | A little bit | Not at all |
| D1 | Introduce you to the knowledge of multiple myeloma disease (e.g., disease characterization, symptom presentation) |  |  |  |  |  |  |  |  |  |  |
| D2 | Introduces you to the main treatment programs and efficacy both domestically and internationally |  |  |  |  |  |  |  |  |  |  |
| D3 | Inform you of knowledge related to the examination of myeloma (e.g., precautions for blood examinations, bone marrow examinations, M proteins, light chains, tumor genes, chromosomes) |  |  |  |  |  |  |  |  |  |  |
| D4 | Assist you in obtaining information about patient's current disease status, future course of treatment and prognosis |  |  |  |  |  |  |  |  |  |  |
| D5 | Informs you of the results about physical examination and guides you in recognizing abnormalities and risk values |  |  |  |  |  |  |  |  |  |  |
| D6 | Inform you of the effects, use method, precautions and common adverse reactions of the drug (e.g., myelosuppression after chemotherapy, peripheral neuropathy, infection) |  |  |  |  |  |  |  |  |  |  |
| D7 | Inform you of the patient's safe activities during hospitalization and after discharge |  |  |  |  |  |  |  |  |  |  |
| D8 | Inform you of the patient's dietary precautions during hospitalization and after discharge |  |  |  |  |  |  |  |  |  |  |
| D9 | Provide knowledge about protecting kidney function |  |  |  |  |  |  |  |  |  |  |
| D10 | Conducting health education activities (e.g., lectures on diseases, distribution of relevant materials) |  |  |  |  |  |  |  |  |  |  |
| D11 | Set up a self-learning online course on multiple myeloma health education |  |  |  |  |  |  |  |  |  |  |
| D12 | Provide you with the process and scope of medical insurance reimbursement |  |  |  |  |  |  |  |  |  |  |
| C1 | Instructs you on ways to assist the patients in turning over |  |  |  |  |  |  |  |  |  |  |
| C2 | Instruct you on routine maintenance of central venous catheters (e.g., PICC, infusion port) |  |  |  |  |  |  |  |  |  |  |
| C3 | Instruct you on ways to prevent, observe, and manage bleeding (e.g., pressure hemostasis and cold compress of nose bleeds) |  |  |  |  |  |  |  |  |  |  |
| C4 | Instruct you to prevent and observe whether blood clots |  |  |  |  |  |  |  |  |  |  |
| C5 | Instruct you on ways to prevent, observe, and manage oral infections (e.g., oral care techniques) |  |  |  |  |  |  |  |  |  |  |
| C6 | Instructs you on ways to prevent, observe, and manage lung infections (e.g., helping pat the patient’s back, instructing the patient in effective coughing and expectoration) |  |  |  |  |  |  |  |  |  |  |
| C7 | Instruct you on ways to prevent, observe, and manage perianal infections (e.g., assisting patients with sitz bath, perianal care) |  |  |  |  |  |  |  |  |  |  |
| C8 | Provides guidance on maintaining the clarity of the patient's bowel movements (e.g., abdominal massage, using softeners) |  |  |  |  |  |  |  |  |  |  |
| C9 | Instruct you on ways to assist patients with exercise and related precautions (e.g., prevention of falls) |  |  |  |  |  |  |  |  |  |  |
| C10 | Instruct you on ways to assist patients with pain relief |  |  |  |  |  |  |  |  |  |  |
| P1 | Guidance on cope with negative emotions such as anxiety, depression, nervousness or irritability |  |  |  |  |  |  |  |  |  |  |
| P2 | Provides you with the skills to communicate with patients |  |  |  |  |  |  |  |  |  |  |
| M1 | Give you advice on raising treatment funds |  |  |  |  |  |  |  |  |  |  |
| M2 | Provides you with the opportunity to communicate with other experienced caregivers |  |  |  |  |  |  |  |  |  |  |
| M3 | The hospital can contact you to follow up with the patient |  |  |  |  |  |  |  |  |  |  |
| M4 | Provide you with contact information for medical and nursing team |  |  |  |  |  |  |  |  |  |  |
| M5 | Medical staff provides online answers to patient questions |  |  |  |  |  |  |  |  |  |  |
